# *IFI16* Can Be Used as a Biomarker for Diagnosis of Renal Cell Carcinoma and Prediction of Patient Survival

**DOI:** 10.3389/fgene.2021.599952

**Published:** 2021-02-15

**Authors:** Baozhong Yu, Xiang Zheng, Zejia Sun, Peng Cao, Jiandong Zhang, Wei Wang

**Affiliations:** ^1^Department of Urology, Affiliated Beijing Chaoyang Hospital of Capital Medical University, Beijing, China; ^2^Department of Clinical Medicine, Capital Medical University, Beijing, China

**Keywords:** *IFI16*, clear cell renal cell carcinoma, biomarkers, diagnosis, prognosis

## Abstract

The incidences of renal cell carcinoma (RCC) increase in number each year and account for about 2–3% of all malignant tumors. Many patients have metastasis by the time of diagnosis, and their prognosis is poor. Therefore, it is essential that new diagnostic and prognostic markers for kidney cancer are identified. In this study, we assessed the potential of *IFI16* as a diagnostic and prognostic marker for RCC. We analyzed the TCGA and UALCAN databases and found *IFI16* to be highly expressed in ccRCC. In addition, high *IFI16* levels positively correlated with lymphatic metastasis, tumor stage, and histopathological grade. Kaplan-Meier curve analysis showed that *IFI16* expression was related to the prognosis of patients, and high *IFI16* expression indicated a worse overall survival (*p* = 5.1E–0.7). Receiver operating characteristic curve analysis showed that a combination of *IFI16* expression and histopathological grade improved predictive accuracy (AUC = 0.697; 95%CI: 0.628–0.765, *P* < 0.001). Finally, the relative levels of *IFI16* in ACHN and Caki-1 cells were higher than that of HK-2 cells by western blotting analysis and RT-PCR. Functional tests showed that knocking down *IFI16* expression inhibited migration and invasion *in vitro*. Therefore, *IFI16* is a potential biomarker for the diagnosis and prognosis of RCC patients.

## Introduction

Renal cell carcinoma (RCC) is a common malignant tumor of the urogenital system, accounting for 80–90% of malignant tumors of the kidney and 2–3% of systemic malignant tumors (Bhatt and Finelli, 2014). The most common histological subtypes include clear cell renal cell carcinoma (ccRCC), papillary renal cell carcinoma and chromophobe renal cell carcinoma. These three subtypes account for more than 90% of all RCCs, and among them, ccRCC accounts for the vast majority (Hsieh et al., 2017). According to previous estimates of the number of patients with ccRCC, the United States will have 73,750 new cases and 14,830 deaths by 2020 (Siegel et al., 2020). Furthermore, ccRCC usually has a poor prognosis because of the high rates of metastasis and mortality (Park et al., 2012; Ascierto et al., 2016). The surgical removal of tumors is an effective treatment for RCC patients. However, because of the mild symptoms and lack of diagnostic markers, approximately 15% of patients have distant metastases at the time of diagnosis (Buti et al., 2013; De Meerleer et al., 2014). Therefore, it is crucial that new diagnostic and prognostic biomarkers are found.

Interferon-inducible protein 16 (*IFI16*) belongs to the HIN-200 protein family and is characterized by a 200-amino-acid motif containing a DNA binding domain at the C-terminus and a PYRIN domain at the N-terminus (Haronikova et al., 2016). *IFI16* is a critical DNA sensor (Singh et al., 2013) for the activation of inflammatory bodies and plays a role in transcriptional regulation (Thompson et al., 2014) and cell proliferation (Xin et al., 2003). The *IFI*16 can bind viral dsDNA and plays an antiviral role by activating the STING-TBK1 pathway to produce IFN-β (Unterholzner et al., 2010). In addition, it can bind with the adaptor molecule ASC and procaspase-1 to form a DNA inflammasome-a cytoplasmic protein complex, which can induce the pyrolysis of inflammatory cytokines and proteolytic maturation (Kerur et al., 2011). However, the mechanism of *IFI16* action in tumor formation is still unclear.

Lin et al. (2017) found that levels of *IFI16* expression were lower in liver cancer tissues compared with adjacent tissues. Forced expression of *IFI16* in liver cancer cells reduced cell viability and promoted apoptosis, and overexpression of *IFI16* inhibited the proliferation of liver cancer cells and reduced the size of tumors. However, Kondo et al. (2012) found that the activation of NF-κB signaling in oral cancer cells promoted the expression of *IFI16*. Moreover, *in vitro* experiments have shown that high expression of *IFI16* promotes cell growth and prevents apoptosis. *IFI16* may play opposite roles in different tumor types, and few studies have explored the biological function of *IFI16* in RCC. Therefore, we aimed to study the potential of *IFI16* as a diagnostic and prognostic marker in RCC.

## Materials and Methods

### Bioinformatics Analysis

The *IFI16* mRNA expression data in the ccRCC organization database were downloaded from the UALCAN dataset. According to the levels of IFI16 expression, the patients were divided into a high-expression group and low-expression group based on the median. The correlations between *IFI16* expression and clinicopathological parameter data (lymph node metastasis, stage, and grade) were analyzed. We used data from the TCGA database to draw a Kaplan-Meier curve to obtain the correlations between *IFI16* expression, overall survival (OS), and disease-free survival (DFS) for RCC, and we verified the prognostic analysis using the ICGC database. The data in the TCGA database were used to draw the ROC curve to determine the suitability of *IFI16* as a biomarker for predicting patient prognosis. The Kaplan-Meier and ROC curves were then employed to analyze the correlation between *IFI16* expression and prognosis.

### RT-PCR

RNA was extracted by using TRIzol (Invitrogen), and RNA was reverse transcribed into cDNA using a reverse transcription kit (TaKaRa RR036B, Japan). The SYBR-Green mixture (TaKaRa RR086B) was subjected to PCR on the ABI Step 1 plus real-time PCR system (Applied Biosystems, United States). GAPDH was used as an endogenous control. The primers for the *IFI16* gene were as follows: Forward: 5’-AACGCTTGAAGACCTGGCTGAA-3’. Reverse: 5’-TTGACAGTGCTGCTTGTGGAGG-3’. The experiments were performed in triplicate.

### Cell Culture

Normal renal tubular epithelial cells, HK-2, and the human RCC cell lines 786-O, ACHN, and Caki-1 were purchased from KeyGEN BioTECH (Jiangsu, China). All cells were cultured in MEM medium containing 10% FBS in a 5% CO_2_ incubator at 37°C. The experiments were performed in triplicate.

### Cell Transfection

We inoculated an appropriate number of cells into a cell culture plate to achieve a cell density of 60–70% during transfection. Then, 5 μL of 20 μM siRNA, normal control (NC), and 5 μL of Lipofectamine 3000 (Invitrogen L3000015, United States) were diluted with 125 μL serum-free medium Opti-MEM and mixed gently. The constructed si-RNA (si-786-O, si-ACHN) sequence and si-NC sequence were transferred into cells using Lipofectamine 3000. Finally, the culture plate was placed in a CO_2_ incubator at 37°C for 48 h. The experiments were performed in triplicate.

### Western Blotting Experiments

The cells were lysed with lysis buffer (Beyotime Biotechnology Institute, China), and the supernatant was collected by centrifugation to obtain the protein. Protein quantification was performed with the BCA kit (Beyotime Biotechnology Institute, China), and proteins were separated by SDS-PAGE. The total protein was then transferred to a nitrocellulose filter membrane. The nitrocellulose filter membrane was placed into a Petri dish, blocking solution containing 5% skimmed milk powder was added, and the dish was placed on a shaker for 2 h. The membrane was transferred to a Petri dish containing the primary antibody and incubated overnight at 4°C with shaking. The next day, the above steps were repeated with the secondary antibody. Finally, G:BOXChemi-XR5 (SYNGENE, United Kingdom) was used for imaging. The experiments were performed in triplicate.

### Cell Counting Kit-8(cck-8) Assay

Cells were digested, counted, and prepared as a cell suspension of 6 × 10^4^ cells/mL, and a 100 μL aliquot of the cell suspension was added to each well of a 96-well cell culture plate. The cell culture plates were cultured for 24, 48, or 72 h. We then added 10 μL CCK-8 to each well of the culture plate and continued culturing for 2 h in a 5% CO_2_ incubator at 37°C. A microplate reader (BioTek ELx800, United States) was used to determine the OD value of each well. The experiments were performed in triplicate and repeated at least three times.

### Wound Scratch Assay

The transfected cells were digested and inoculated into a six-well plate. After the cells adhered to the wall, 0.25% trypsin was added to collect the cells, which were inoculated into a fresh six-well plate. When the cell aggregation reached about 80%, pipette tips were used to draw an even line in the six-well plate. The floating cells were washed with PBS, fresh culture medium was added, and the culture was placed in a 5% CO_2_ incubator at 37°C to continue culturing. After culturing for 24 h, the cells were photographed to measure the cell migration distance. The experiments were performed in triplicate.

### Transwell Assay

We placed 100 μL of each cell suspension (si-786-O, si-NC, si-ACHN, si-NC) into transwell chambers (Corning Incorporated, United States), and then added 500 μL of FBS-containing medium to the lower chamber. The 24-well cell culture plate was placed into a 37°C, 5% CO_2_ incubator for 24 h. Finally, 500 μL of 0.1% crystal violet was added to the 24-well plate; the plate was placed in the incubator, and the membrane immersed in the dye. Then take it out at 37°C for 30 min. Transwell chambers with or without Matrigel (BD, United States) were used to assess the invasion or migration characteristics of the cells, as observed with a digital camera system. The experiment was repeated at least three times. The experiments were performed in triplicate.

### Statistical Analysis

Statistical analysis was performed using SPSS statistical software and Graphpad Prism 7.0. Pearson’s chi-square test was used to analyze the correlations between *IFI16* expression levels and clinicopathological parameters of ccRCC. Univariate and multivariate Cox regression analyses were used to examine the significance of IFI16 expression for patient survival. A Kaplan-Meier curve was used to analyze the correlation between the expression level of *IFI16* and the prognosis of ccRCC patients. We employed ROC curves of *IFI16* expression, tumor grade, and the two combined, to evaluate the predictive efficiency of *IFI16* for ccRCC patients, and the area under the curve was obtained. The *P*-value was calculated using the unpaired *t*-test. A *P*-value of < 0.05 was considered statistically significant.

## Results

### *IFI16* Is Significantly Highly Expressed and Is Related to Clinical-Pathological Parameters in ccRCC Tissues

**FIGURE 1:**
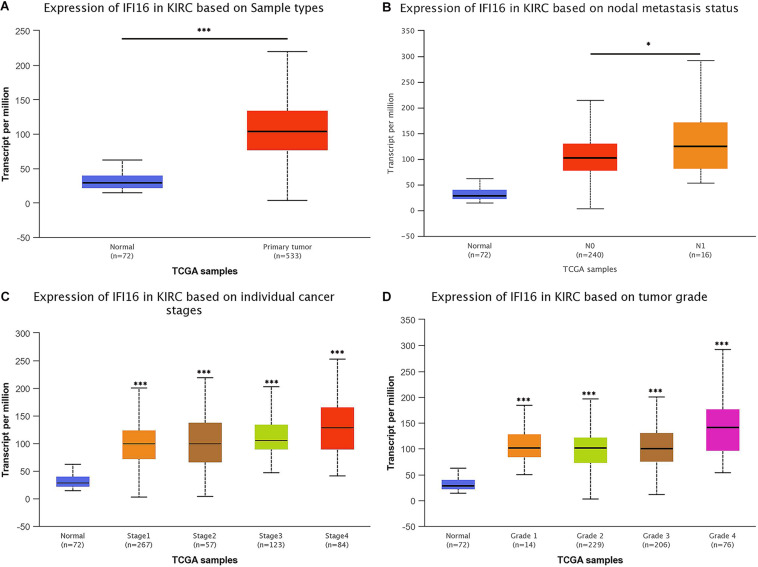
*IFI16* mRNA expression and relationship with tumor stage, lymphatic metastasis, and histopathological grade in ccRCC (UALCAN). **(A)** Relative expression of *IFI16* in ccRCC and normal tissues. **(B)** Relationship between *IFI16* expression and lymphatic metastasis in ccRCC. **(C)** Relationship between *IFI16* expression and tumor stage in ccRCC. **(D)** Relationship between *IFI16* expression and histopathological grade in ccRCC. *P < 0.05, ***P < 0.001.

From the UALCAN database, we found that the expression of *IFI16* in ccRCC patients (*n* = 533) was significantly upregulated compared with expression in the adjacent tissues (*n* = 72) (Figure 1A). Next, we evaluated the relationship between expression levels of *IFI16* and clinical-pathological parameters (lymph node metastasis, stage, and grade) in ccRCC patients (Figures 1B–D): *IFI16* was highly expressed in ccRCC patients with lymph node metastasis, higher tumor stages, and higher histopathological grades.

### T Relationship Between *IFI16* Expression and Prognosis of ccRCC Patients

**FIGURE 2:**
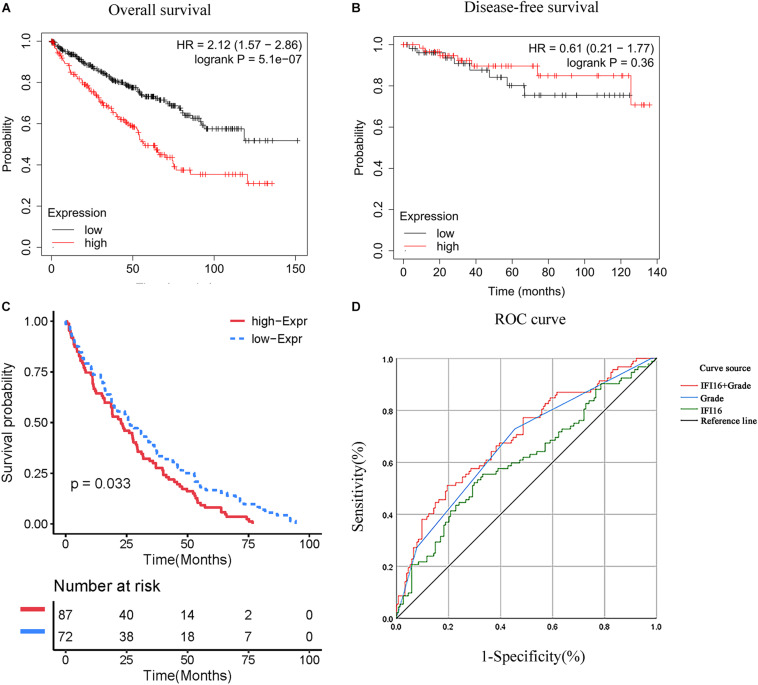
Correlation between *IFI16* expression and prognosis. **(A)** Correlation between *IFI16* expression and OS, as shown by Kaplan-Meier curve. **(B)** Correlation between *IFI16* expression and DFS, as shown by Kaplan-Meier curve. **(C)** Correlation between *IFI16* expression and OS (ICGC database). **(D)** Use of *IFI16* expression and histopathological grade to evaluate the predictive efficiency of ccRCC patients using a ROC curve.

We used the ccRCC data in the TCGA database to draw the Kaplan-Meier curve. Kaplan-Meier curve analysis showed that the expression of *IFI16* was significantly related to the prognosis of patients, and the higher the expression, the worse the OS rate of patients was (Figure 2A). However, expression levels of *IFI16* were not associated with the DFS of patients with ccRCC (Figure 2B) and were not statistically significant. We verified the prognostic analysis using the ICGC database (Figure 2C). Increased levels of *IFI16* expression, were associated with increasingly worse OS (*P*< 0.033). In the univariate analysis (Table 1), patient survival was not related to sex. However, it was significantly associated with tumor stage (I + II vs. III + IV: HR = 3.676, 95%CI = 2.366–5.711, *P* = 7.05E–09), histopathological grade (G1 + 2 vs. G3 + 4: HR = 2.629, 95%CI = 1.655–4.176, *P* = 4.26E–05), and *IFI16* expression level (low vs. high: HR = 1.043, 95%CI = 1.024–1.063, *P* = 1.07E–05). In the multivariate analysis, age, grade, M stage, and *IFI16* expression were significantly correlated with patient survival (low vs. high: HR = 1.023, 95% CI = 1.002–1.045, *P* = 0.032).

**TABLE 1:** *IFI16* expression and survival of ccRCC patients.

Variable	Univariate analysis		Multivariate analysis	
	HR (95% CI)	*P*-value	HR (95% CI)	*P*-value
Age (≤60 vs. >60 years)	1.546 (1.011–2.362)	0.044	1.632 (1.058–2.520)	0.027
Gender (Female vs. male)	1.013 (0.666–1.541)	0.951	1.040 (0.668–1.618)	0.863
Grade (G1 + G2 vs. G3 + G4)	2.629 (1.655–4.176)	4.26E–05	1.749 (1.066–2.868)	0.027
Stage (I + II vs. III + IV)	3.676 (2.366–5.711)	7.05E–09	1.417 (0.559–3.594)	0.463
T (T1 + T2 vs. T3 + T4)	3.311 (2.167–5.058)	3.07E–08	1.476 (0.642–3.394)	0.359
M (M0 vs. M1)	4.073 (2.634–6.300)	2.76E–10	2.297 (1.358–3.885)	0.002
N (N0 vs. N1)	2.932 (1.516–5.668)	1.00E–03	1.394 (0.686–2.830)	0.358
IFI16 (low vs. high)	1.043 (1.024–1.063)	1.07E–05	1.023 (1.002–1.045)	0.032

### Predictive Efficiency of *IFI16*

ROC curves for *IFI16* expression, histopathological grade, and the combination of the two were used to evaluate the predictive efficiency of ccRCC patients with *IFI16*. *IFI16* expression alone could be used to evaluate the predictive efficiency of patients; the AUC was 0.608 (95% CI: 0.534–0.682, *P* = 0.002), and the sensitivity and specificity were 55.4 and 66.9%, respectively. In addition, the histopathological grade alone could be used to evaluate the predictive efficiency of patients; the AUC was 0.673 (95% CI: 0.603–0.743, *P* < 0.001), and the sensitivity and specificity were 72.8 and 54.5%, respectively. However, the combination of *IFI16* expression and histopathological grading improved prediction accuracy; the AUC was 0.697 (95% CI: 0.628–0.765, *P* < 0.001), and the sensitivity and specificity were 51.1 and 80.5%, respectively.

### Expression Levels of *IFI16* Were Analyzed in RCC Cell Lines

**FIGURE 3:**
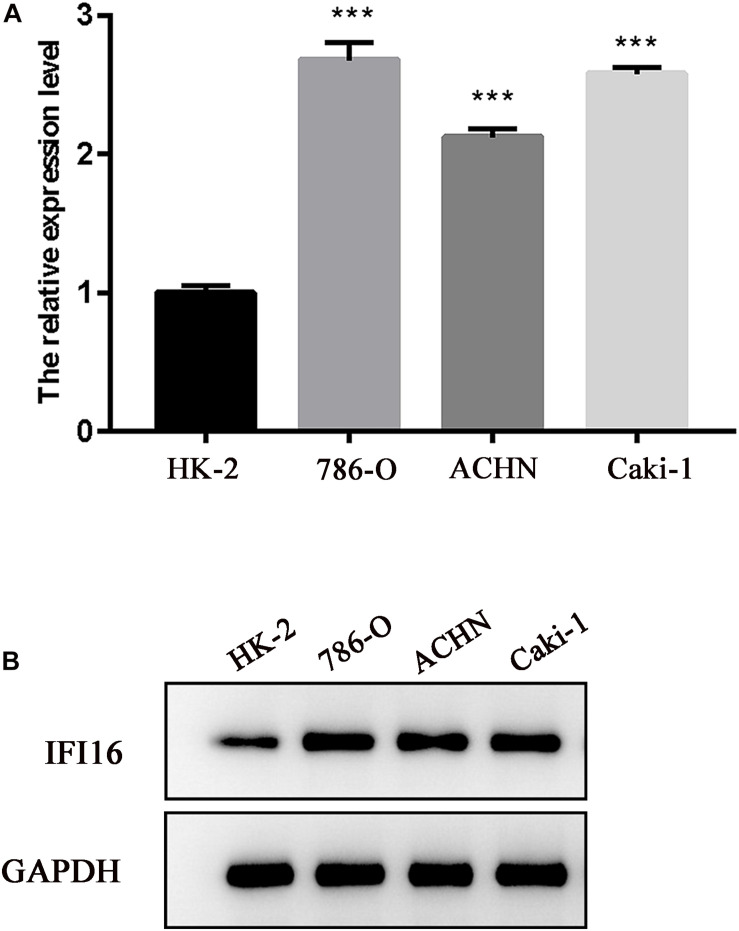
Expression levels of *IFI16* in RCC cells. **(A)** Western blot analysis of *IFI16* expression levels in RCC cell lines and normal renal epithelial cells. **(B)** RT-PCR analysis of *IFI16* expression levels in RCC cell lines and normal renal epithelial cells. ***P < 0.001.

To verify the results of the UALCAN and TCGA databases, western blot and RT-PCR analyses of RCC cell lines were performed for *IFI16*. Compared with normal renal epithelial cells (HK-2), *IFI16* was more highly expressed in the RCC cell lines (Figures 3A,B).

### Transfection and CCK-8 Assay

**FIGURE 4:**
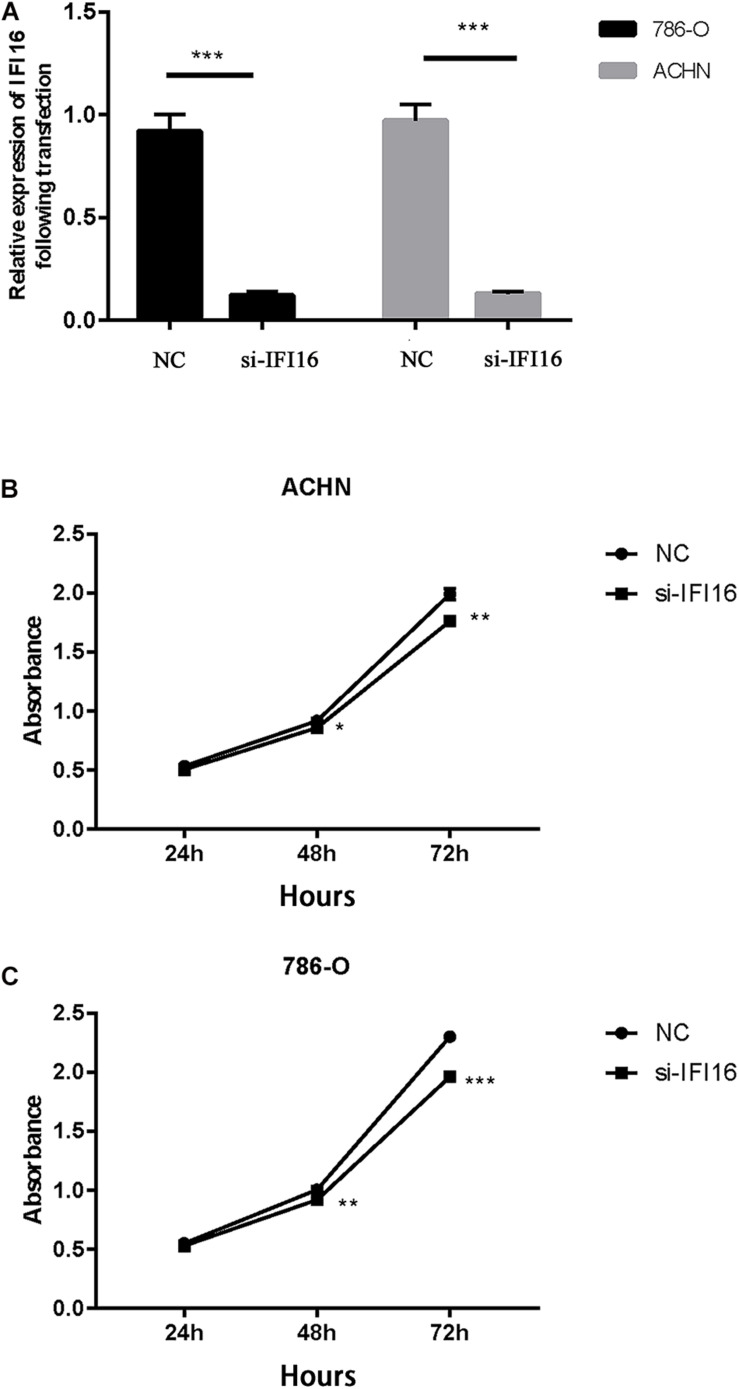
Transfection and CCK-8 assay. **(A)** Relative transfection efficiency of si-IFI16 compared with NC according to RT-PCR. **(B)** Cell proliferation assay of 786-O cells. **(C)** Cell proliferation assay of ACHN cells. *P < 0.05, **P < 0.01, ***P < 0.001.

First, we transfected the si-IFI16 knockdown plasmid and the corresponding si-NC into renal RCC cells (786-O and ACHN cells), resulting in low *IFI16* expression. The relative transfection efficiencies of si-IFI16 and NC were verified by real-time PCR. The *IFI16* expressions of 786-O and ACHN cells transfected with si-IFI16 were 0.12 ± 0.02 and 014 ± 0.01, respectively, while the expressions of RCC cells transfected with NC were 0.92 ± 0.08 and 0.94 ± 0.14 (Figure 4A). We then performed CCK-8 analysis to study the effect of *IFI16* on cell proliferation. The results showed that knockdown of *IFI16* inhibited the proliferation of RCC cells (Figures 4B,C).

### Knockdown of *IFI16* Inhibited the Migration of RCC Cells in the Wound Scratch Assay

**FIGURE 5:**
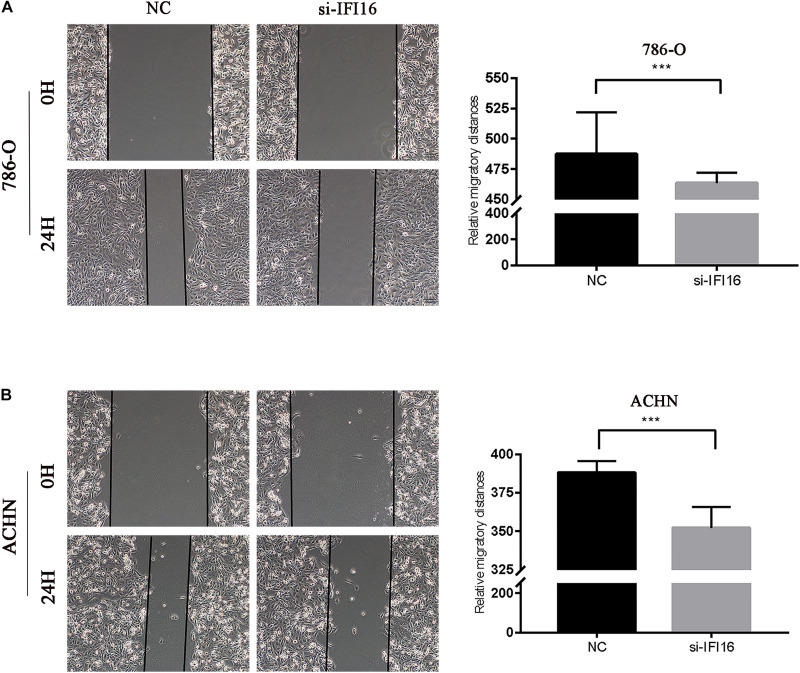
Wound scratch assay results showing inhibition of *IFI16*-knockdown RCC cell migration. **(A,B)** Wound scratch assay of 786-O and ACHN cells. Magnification 100×. ***P < 0.001.

We conducted the wound scratch assay to study the effect of *IFI16* on the migration of RCC cells (786-O and ACHN cells). The migration distance of the NC group was significantly longer than that of the si-IFI16 group for both 786-O or ACHN cells (Figures 5A,B).

### Knockdown of *IFI16* Inhibited the Migration and Invasion of RCC Cells in the Transwell Assay

**FIGURE 6:**
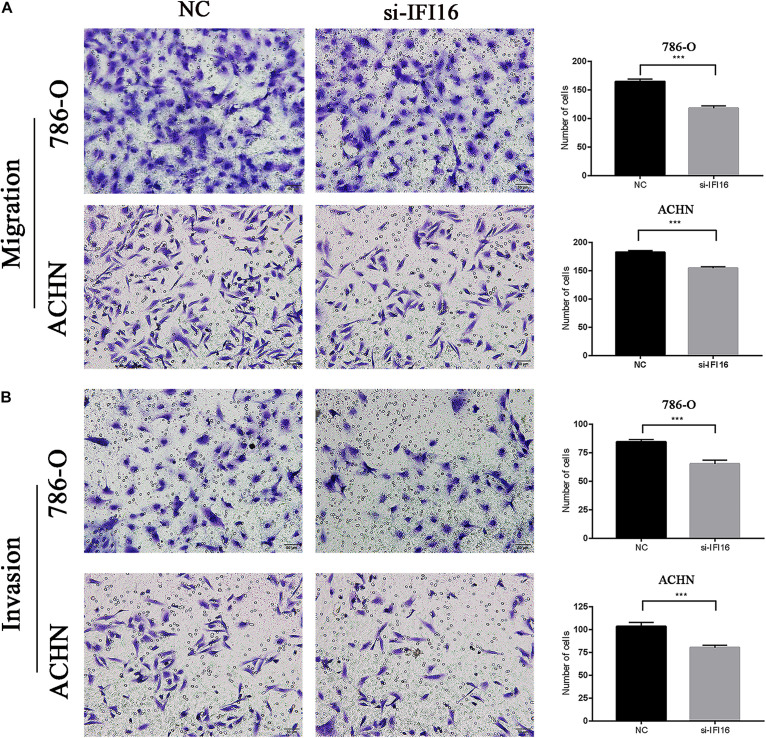
Transwell assay results showing inhibition of *IFI16*-knockdown RCC cell migration and invasion. **(A,B)** Migration assay of 786-O and ACHN cells. Relative migratory cells per field in 786-O and ACHN cells transfected with si-IFI16 and NC. **(B)** Invasion assay of 786-O and ACHN cells. Relative invasion cells per field in 786-O and ACHN cells transfected with si-IFI16 and NC. ***P < 0.001.

We analyzed whether *IFI16* affects the migration and invasion of RCC cells (786-O and ACHN cells) in the transwell assay. The results showed that the si-IFI16 group had fewer cells than the NC group. Therefore, the knockdown of *IFI16* reduced the migration and invasion abilities of the 786-O and ACHN cells (Figures 6A,B). The wound scratch and transwell assays showed that kidney cancer cells with high *IFI16* expression migrated and invaded faster than cells with low expression.

## Discussion

RCC is the most common type of upper urinary tract tumor, and the incidence of RCC has gradually increased in the past 20 years (Saad et al., 2019). The early stages of RCC are mostly asymptomatic and are usually found on unrelated imaging studies. In approximately 16% of patients, the tumor has already metastasized by the time RCC has been diagnosed (Siegel et al., 2020). Therefore, if diagnosed early, the prognoses for RCC patients are significantly improved. At present, there is no accurate biomarker for the early diagnosis and prognosis of kidney cancer.

*IFI16* is a member of the interferon-inducible p200-protein family (Gariglio et al., 2011), and multiple studies have shown that *IFI16* plays opposite roles in different types of tumors (He et al., 2017). For example, De Andrea et al. (2007) found that the downregulation of *IFI16* may lead to the proliferation and tumorigenesis of head and neck squamous cell carcinoma cells. Whereas, Zhang et al. (2007) confirmed that the overexpression of *IFI16* in human osteosarcoma and chondrosarcoma cell lines inhibited cell proliferation and colony formation. When the expression of *IFI16* in tumor cells is knocked down, this effect is reversed. The effects of *IFI16* are related to p21, cyclin E, cyclin D1, c-Myc, and Ras. Lin et al. (2017) showed that overexpression of *IFI16* inhibited colony formation and cell proliferation; moreover, cell apoptosis increased and migration ability was impaired. Mechanistically speaking, *IFI16* upregulates the levels of p21WAF1/CIP1 by activating p53 via Ser15, thereby inhibiting tumor growth, migration, and invasion. However, Cai et al. (2020) found that high levels of *IFI16* expression in cervical cancer upregulate the STING-TBK1-NF-kB pathway. In the immune microenvironment, PD-L1 production promotes the progression of cervical cancer. In a study by Kondo et al. (2012), high expression of *AIM 2* and *IFI16* inhibited apoptosis and promoted the proliferation of oral squamous cell carcinoma cells. Therefore, *IFI16* may play a role in promoting or inhibiting cell proliferation, migration, and invasion in tumors. However, there are no reports on how *IFI16* affects the characteristics of RCC cells.

In this study, we used the UALCAN database to analyze the expression of *IFI16* in ccRCC patients (Figure 1) and found that *IFI16* was highly expressed in cancer tissues compared to normal tissues. Bioinformatic analysis showed that the expression levels of *IFI16* were related to clinicopathological parameters: *IFI16* was highly expressed in ccRCC patients with lymph node metastasis, higher tumor stages, and higher histopathological grades. The Kaplan-Meier survival curve showed that high expression of *IFI16* was significantly associated with a poor OS, and the results of Cox regression analysis confirmed this. Through ROC analysis, *IFI16* expression and histopathological grade could be used to predict the prognosis of patients to some degree, and when the two aspects were combined the prediction accuracy increased to a certain extent. To verify the accuracy of the database, we performed western blot analysis and RT-PCR. Compared with HK-2 cells, there was high *IFI16* expression in RCC lines (786-O and ACHN), which is consistent with the database results. The CCK-8 analysis suggested that knocking down *IFI16* expression in the renal cancer cell line inhibits the proliferation of RCC cells. In the wound scratch assay, the migration distance of the NC group was longer than that of the si-IFI16 group, which showed that the knockdown of *IFI16* reduced the migration ability of 786-O and ACHN cells. The transwell assay showed that there were fewer cells in the si-IFI16 group than in the NC group, whether it was 786-O or ACHN cells. Together, these results confirmed that *IFI16* promotes the migration and invasion of RCC cells.

There were some drawbacks to our study. First, the mechanism of *IFI16* overexpression and the molecular mechanism by which *IFI16* promotes RCC progress remain unclear. Second, we used cancer cell lines for verification and did not collect clinical specimens, thus further research is needed. We aim to continue to explore the mechanisms of *IFI16* carcinogenesis in follow-up studies to provide a theoretical basis for IFI16-targeted therapy for RCC patients. Finally, serum and urine specimens are readily available, and future research using these samples should be valuable.

## Conclusion

In conclusion, we confirmed that *IFI16* is highly expressed in RCC and is associated with patient prognosis. In addition, *IFI16* may be a biomarker for RCC diagnosis and prognosis. *IFI16* targeted therapy may provide new directions and strategies for RCC therapy.

## Data Availability Statement

The original contributions presented in the study are included in the article/supplementary material, further inquiries can be directed to the corresponding author/s.

## Author Contributions

ZS, PC, JZ, and WW conceived and supervised the study. BY and XZ wrote the manuscript. All authors read and approved the final manuscript.

## Conflict of Interest

The authors declare that the research was conducted in the absence of any commercial or financial relationships that could be construed as a potential conflict of interest.

## Abbreviations

*IFI16*: interferon-inducible protein 16
RCC: renal cell carcinoma
ROC: Receiver operating characteristic curve
ccRCC: clear cell renal cell carcinoma
DFS: disease-free survival
OS: Overall survival
KIRC: Kidney renal clear cell carcinoma.
